# The effect of X-rays and C-ions on pluripotent embryonic stem cells

**DOI:** 10.1093/jrr/rrt175

**Published:** 2014-03

**Authors:** Sabine Luft, Diana Pignalosa, Onetsine Arrizabalaga, Elena Nasonova, Alexander Helm, Marco Durante, Sylvia Ritter

**Affiliations:** 1 GSI Helmholtzzentrum für Schwerionenforschung GmbH, Planckstraße 1, 64291 Darmstadt, Germany; 2 Joint Institute for Nuclear Research, Dubna, Russia; 3 Technical University of Darmstadt, Germany

**Keywords:** embryonic stem cells, pluripotency, genomic integrity

## Abstract

Embryonic stem cells (ESC) are characterized by both the capacity of infinite self-renewal and the ability to give rise to all the three germ layers emphasizing the need to strictly control the genetic integrity. To date, ESC are a powerful tool in disease modeling, tissue engineering and drug testing. However, in the field of radiation research, their potential has not been exploited.

We used the mouse ESC line D3 as a model to examine the effects of X-rays or C-ions (spread out Bragg peak, energy 106–147 MeV/u, average LET = 75 keV/µm) [ 1]. Doses of 0.5–5 Gy were applied and endpoints such as cell cycle progression (measured by flow cytometry), apoptosis (microscopic analysis of cell nucleus morphology), induction of chromosome aberrations (mFISH analysis), presence of pluripotency markers Oct3/4 and SOX2 (western blotting) and differentiation capacity by means of an embryoid body formation assay were analyzed up to 17 days post-irradiation. The experiments show that cells undergo a transient G_2_ arrest following exposure. After G_2_ checkpoint release, an increase in the apoptotic index is observed for both radiation types (3.7-fold increase for 2 Gy X-ray and 2.4-fold increase for 2 Gy C-ions). C-ions induce more structural chromosomal aberrations in first cycle cells than X-rays. During subsequent cell divisions, the frequency of chromosome aberrations declines: After >7 population doublings (8 days after exposure), the aberration frequency in the progeny of X-ray exposed cells returns to the control level (7% aberrant cells), while the progeny of C-ion exposed cells still harbor significantly more aberrations than control cells, which is mainly due to transmissible translocations.

The expression of pluripotency markers is maintained in cells surviving X-ray or C-ion exposure. This finding is supported by examining the differentiation capacity of ESC through the formation of embryoid bodies. Our experiments show that after X-ray or C-ion exposure, cells are able to develop spontaneous beating activity, indicating the differentiation ability into mesodermal cell lineages, i.e. beating cardiomyocytes. However, following C-ion exposure, the formation of beating clusters was delayed compared with control cells.

Moreover, our chromosome studies revealed that unexposed cells carry a high frequency of numerical aberrations. These comprise trisomies of chromosome 8 and 11 with a frequency of 29 ± 8% and 26 ± 6% respectively, as well as nullisomy of chromosome Y with a frequency of 35 ± 3%. Aneuploidy is a typical feature of mouse ESC and has been related to cell culture methods [ 2] and passage number. Because aneuploidy may affect gene expression and influence the properties of a cell population, the relevance of experiments based on mouse ESC is limited.

To overcome this problem, we recently extended our studies to human ESC. Human ESC are known to be cytogenetically more stable than mouse ESC, and represent a model that is closer to human embryonic development. Indeed, first investigations revealed a lower faction of cells with numerical and structural aberrations in the human ESC line H9 [ 3] compared with the mouse ESC line D3 (2% vs. 73% and 3% vs. 7%, respectively).
